# Beyond Biodiversity: Fish Metagenomes

**DOI:** 10.1371/journal.pone.0022592

**Published:** 2011-08-04

**Authors:** Alba Ardura, Serge Planes, Eva Garcia-Vazquez

**Affiliations:** 1 Department of Functional Biology, University of Oviedo, Oviedo, Spain; 2 EPHE-URA CNRS 1453, Université de Perpignan, Perpignan Cedex, France; 3 USR 3278 CNRS, EPHE Centre de Recherche Insulaire et Observatoire de l'Environnement (CRIOBE) BP, Papetoai, Moorea, Polynésie française; Argonne National Laboratory, United States of America

## Abstract

Biodiversity and intra-specific genetic diversity are interrelated and determine the potential of a community to survive and evolve. Both are considered together in Prokaryote communities treated as metagenomes or ensembles of functional variants beyond species limits.

Many factors alter biodiversity in higher Eukaryote communities, and human exploitation can be one of the most important for some groups of plants and animals. For example, fisheries can modify both biodiversity and genetic diversity (intra specific). Intra-specific diversity can be drastically altered by overfishing. Intense fishing pressure on one stock may imply extinction of some genetic variants and subsequent loss of intra-specific diversity. The objective of this study was to apply a metagenome approach to fish communities and explore its value for rapid evaluation of biodiversity and genetic diversity at community level.

Here we have applied the metagenome approach employing the Barcoding target gene COI as a model sequence in catch from four very different fish assemblages exploited by fisheries: freshwater communities from the Amazon River and northern Spanish rivers, and marine communities from the Cantabric and Mediterranean seas.

Treating all sequences obtained from each regional catch as a biological unit (exploited community) we found that metagenomic diversity indices of the Amazonian catch sample here examined were lower than expected. Reduced diversity could be explained, at least partially, by overexploitation of the fish community that had been independently estimated by other methods.

We propose using a metagenome approach for estimating diversity in Eukaryote communities and early evaluating genetic variation losses at multi-species level.

## Introduction

Biodiversity found on Earth today is the result of 3.5 billion years of evolution. There are varied definitions of biodiversity, from “the totality of genes, species, and ecosystems of a region” to “the diversity of genes and organisms”. In 1992 the Conference of the United Nations on Environment and Development was celebrated in Rio de Janeiro, Brazil, also known as the “Summit of the Earth”. In this meeting the Convention on Biological Diversity (CDB), which focused on the conservation and the sustainable use of the biodiversity, was signed. CDB has three main goals: conservation of biodiversity, sustainable use of its components and fair equitable sharing of benefits arising from genetic resources. Ensuring Environmental Stability is one of the United Nations Millennium Development Goals established to end poverty, showing the great importance of the environment and biodiversity. Biodiversity is commonly referred to as the combination of species present in an ecosystem. Each species within an ecosystem exhibits in addition to other types of diversity, intra-specific genetic diversity. Genetic diversity is thus a crucial component of biodiversity and fundamental to species survival and to enabling appearance of new species. It is the basis of reproductive performance, resistance to diseases and capacity of adaptation to environmental changes [1]–[3]. Biodiversity and genetic diversity are dependent upon each other: diversity within a species is necessary to maintain diversity among species, and vice versa [4]. We thus infer that when ecosystems are subjected to exploitation or other alterations, complete estimates that combine both types of diversity are crucial for describing community conservation status.

The concept of metagenome includes both inter- and intra-specific genetic diversity because all the individual genomes present in an environmental sample are considered as a unit. Metagenomics is the culture-independent genomic analysis of a community of microorganisms, generally aimed at community-wide assessment of metabolic functions [5]. The analysis of metagenomic data provides a way to identify new organisms and isolate complete genomes from uncultured species that are present within an environmental sample [6]. Therefore, a metagenome could also be defined as an assemblage of genomes that occupies an ecological niche. To date, the metagenome concept is reserved for microorganisms: ruminant metagenome [7], marine metagenome [8], metazoan metagenome [9] etc. (http://www.ncbi.nlm.nih.gov/Genbank/metagenome.html). Although it is difficult to extend the perspective to higher eukaryotes like vertebrates, given the huge size of some genomes, a shortcut is possible if we focus on one or a few genes. The international Barcoding initiative [10] can help in this task. DNA Barcoding is based on the use of a standard region employed to catalogue the world's biota, including fish: FISH-BOL is the campaign aimed at DNA barcoding all fish species [11] (http://www.fishbol.org/). The mitochondrial COI gene targeted by Barcoding projects is useful for species identification, and also exhibits intra-specific polymorphism. Within-species genetic diversity being crucial for this approach, COI intra-specific variation could be advantageous over more conserved genes like the 18S rRNA, which has been employed in metagenome approaches applied to lower Eurkaryotes [9]. The typical sequence information gathered for DNA barcoding can provide an early insight into the patterning of genomic diversity within a species, facilitating comparative studies of genetic diversity in different species or ecological settings [12].

Fisheries have been identified as one of the main causes for the loss of animal genetic diversity [13]. Fisheries exploit diversity, both species biodiversity and intra-specific diversity. Most targeted species are predators which as a consequence are declining dramatically and altering the rest of species in the trophic chain [14], [15]. Intra-specific diversity can also be drastically altered by fisheries. For example, changes in life histories from the systems results in remaining breeders becoming increasingly smaller [16], [17]. On the other hand, overfishing on one stock may imply extinction of some genetic variants and subsequent loss of intra-specific diversity, with unpredictable effects on species biodiversity [4].

The tropical Amazon River is an ecologically critical reservoir of Earth diversity [18]. Its fish community is exploited by artisanal fisheries and basic control tools are being developed now [19]. Recent population declines of targeted species [20] suggest fisheries overexploitation, and could be taken as a warning to assess the current levels of genetic diversity of the fish community and, if necessary, rapidly address conservation measures. Our objective being a rapid evaluation of diversity of the exploited species, we have analyzed a sample of commercial fish representing 65% of the Amazonian catch (in annual tons) in the central region of Manaus (Table 1), applying a metagenome perspective with Barcoding sequences as a tool. To our knowledge this is the first time this procedure has been followed in fishery science. To expand this idea with other contrasting case studies we have chosen three different well known fisheries from European regions, one continental (freshwater) and two marine. The freshwater fishery practiced in north Spanish rivers is sportive (angling) and strongly targeted on Salmonids. The two marine areas considered were: the Mediterranean biodiversity hotspot where populations are also declining [21], [22] (Roussillon south French area); the Cantabric region, where fisheries target large predators similar to most marine commercial fisheries worldwide [15] (north Spanish Atlantic area near the Bay of Biscay).

**Table 1:** Fish catches in the four case studies considered.

AMAZON RIVER				MEDITERRANEAN SEA				CANTABRIC SEA				SPANISH RIVERS			
Species (Common name)	tons/year	%	Σ %	Species (Common name)	tons/year	%	Σ %	Species (Common name)	tons/year	%	Σ %	Species (Common name)	tons/year	%	Σ %
**Jaraqui**	16,086	26.67	26.67	**Sardine**	13,188	43.30	43.30	**Mackerel**	5,405.35	32.63	32.63	**Brown trout**	348.6	88.4	88.84
**Curimata**	6,934	11.50	38.17	**Anchovy**	4,184	13.74	57.04	**Sardine**	2,725.52	16.45	49.08	**Atlantic salmon**	9.2	2.3	**90.73**
**Pacu**	6,543	10.85	49.03	**Mackerel**	1,659	5.45	62.49	**Blue whiting**	2,042.49	12.33	61.41	European eel	8	2.03	92.76
**Piramutaba**	3,794.5	6.30	55.33	**Hake**	1,331	4.37	**66.86**	**Tuna**	1,727.15	10.43	**71.84**	Shads	Miscellaneous		
**Tambaqui**	2,664.5	4.42	59.75	Atlantic mackerel	616	2.02	68.88	Hake	1,625.82	9.81	81.65	Common carp	Miscellaneous		
**Matrinxa**	2,394.5	3.97	63.72	European seabass	279	0.92	69.80	Atlantic mackerel	482.35	2.91	84.56	Rainbow trout	Miscellaneous		
**Sardinha**	2,316	3.84	**67.56**	Gilt-head bream	248	0.81	70.61	Frog-fish	298.25	1.80	86.36	Brook trout	Miscellaneous		
Mapara	2,292	3.80	71.36	Chub mackerel	106	0.35	70.96	Conger eel	268.17	1.62	87.98	Gudgeon	Miscellaneous		
Tucunare	2,269.5	3.76	75.12	Blue whiting	14	0.05	71.01	Red mullet	157.80	0.95	88.93	Iberian nase	Miscellaneous		
Pirapichinga	1,928	3.20	78.32	Black seabream	9	0.03	71.04	Megrim	136.10	0.82	89.75	Common minnow	Miscellaneous		
Others	13,084	21.68	100	Others	8,825	28.93	100	Others	1,696.80	10.25	100	Others	28.40	7.20	100
**TOTAL**	60,306	100		**TOTAL**	30,459	100		**TOTAL**	16,565.8	100		**TOTAL**	394.2	100	

Species are ordered by annual catch tons. The percent and cumulative percent of each species over total catch are presented for each fishery. In bold, species comprising 65% catch (>90% in Spanish rivers).

## Results

The Amazon River sample contained more species (seven) than samples from the Mediterranean and Cantabric marine fisheries (four species each), whereas Spanish rivers samples only contained two species (Table 1). The DNA sequences obtained for all species were submitted to the GenBank (http://www.ncbi.nlm.nih.gov) and are available with the accession numbers shown in Table 2. The average trophic level of the catch (Table 3) was lower in the Amazon River than in the other locations (2.636 versus 3.058, 3.719 and 3.738 respectively). The species in our samples represented 0.56% to 12% of the total number of fish species inventoried from the respective ecosystems (Table 3).

**Table 2:** Fish species analyzed in each region: common name; specific name; n° haplotypes of each species; region; GenBank Accession Number.

COMMON NAME	SPECIFIC NAME	N° HAPLOTYPES	REGION	GenBank A.N.
Anchovy	*Engraulis encrasicolus*	5	Mediterranean Sea (Roussillon, France)	HM480814–HM480818
Atlantic salmon	*Salmo salar*	1	Spanish rivers (Asturias, Spain)	HM480828
Blue whiting	*Micromesistius poutassou*	4	Cantabric Sea (Asturias, Spain)	HM480790–HM480792; HQ340605
Brown trout	*Salmo trutta*	3	Spanish rivers (Asturias, Spain)	HM480829–HM480831
Curimata	*Prochilodus nigricans*	4	Amazon River (Manaus, Brazil)	FJ418758; HM480806–HM480808
Hake	*Merluccius merluccius*	2	Mediterranean Sea (Roussillon, France)	HM480820–HM480821
Jaraqui	*Semaprochilodus insignis*	4	Amazon River (Manaus, Brazil)	FJ457765; HM480809–HM480811
Mackerel	*Scomber scombrus*	1	Mediterranean Sea (Roussillon, France)	HM480819
		5	Cantabric Sea (Asturias, Spain)	HM480796–HM480800
Matrinxa	*Brycon melanopterus*	1	Amazon River (Manaus, Brazil)	FJ978040
Pacu	*Mylossoma duriventre*	1	Amazon River (Manaus, Brazil)	HM453212
Piramutaba	*Brachyplatystoma vaillantii*	1	Amazon River (Manaus, Brazil)	HM453213
Sardine	*Sardina pilchardus*	7	Mediterranean Sea (Roussillon, France)	HM480822–HM480827; HQ340604
		5	Cantabric Sea (Asturias, Spain)	HM480801–HM480805
Sardinha	*Triportheus elongatus*	1	Amazon River (Manaus, Brazil)	GU060427
Tambaqui	*Cichla temensis*	2	Amazon River (Manaus, Brazil)	HM480812–HM480813
Tuna	*Thunnus alalunga*	3	Cantabric Sea	HM480793–HM480795

**Table 3:** Genetic diversity and biodiversity in the four case studies considered.

	AMAZON RIVER	MEDITERRANEAN SEA	CANTABRIC SEA	ASTURIAN RIVERS
Number of Barcoding haplotypes	14	15	17	4
COI polypeptide variants	7	5	4	1
Haplotypes/polypeptides	2	3	4.25	4
Haplotypic diversity (standard deviation)	0.903 (0.032)	0.829 (0.063)	0.919 (0.028)	0.613 (0.067)
Nucleotide diversity (standard deviation)	0.145 (0.071)	0.195 (0.095)	0.222 (0.108)	0.048 (0.024)
Fish species representing 65% catch/inventoried in the ecosystem	7/1218	4/713	4/148	2/17
Mean trophic level (variance)	2.636 (0.727)	3.508 (0.417)	3.719 (0.314)	3.738 (0.813)
Shannon Index (H)	1.59	0.9904	1.329	0.1985
Taxonomic Index (TTD)	344.4	244.4	244.4	33.3
Phylogenetic Index (sΦ^+^)	350	266.7	266.7	116.7

For the analyzed samples: number of haplotypes; number of cytochrome oxydase I (COI) polypeptide variants; ratio between them (haplotypes/polypeptides); haplotypic diversity (Hd); nucleotide diversity (π).

For fish species representing 65% of the catch in each region: mean trophic level; ecological diversity index (Shannon); taxonomic index (Total Taxonomic Diversity); phylogenetic index (total sΦ^+^ value).

**Figure 1:**
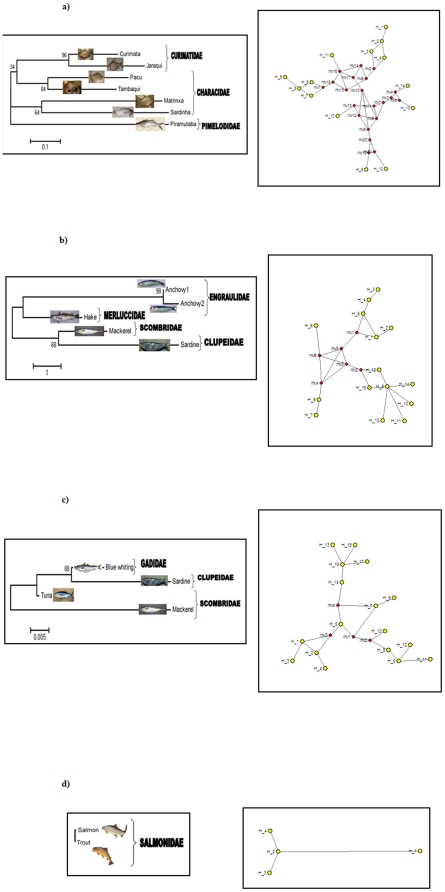
At right (R), haplotype networks obtained for the COI gene from Amazonian (a), Mediterranean (b), Cantabric (c) and Spanish freshwater (d) fish. At left (L), phylogenetic trees constructed based on COI protein sequences of the same samples. In the haplotype networks, yellow dots correspond to real haplotypes and red dots are internal nodes representing hypothetical intermediate mutations. For Spanish freshwater fish a phylogenetic tree cannot be constructed because the protein sequence is identical for the two species (*Salmo trutta* and *Salmo salar*).

Considering all Barcoding sequences obtained from each catch as a unit –the metagenomic diversity-, the highest number of haplotypes (and accordingly hyplotypic diversity) and nucleotide diversity corresponded to the Cantabric catch, followed by the Mediterranean, Amazonian and Spanish rivers catches (Table 3; 17, 15, 14 and 4 haplotypes respectively, represented as yellow dots in Fig. 1R). From this perspective, the Amazonian catch, although containing more species, exhibited only moderate metagenomic diversity.

From the point of view of final gene products (polypeptides), because the COI protein is highly conserved, intraspecific diversity was mostly due to synonymous substitutions. Accordingly (Fig. 1L), only one putative protein was obtained per species (per genus in the case of Atlantic salmon *Salmo salar* and brown trout *S. trutta*, with identical proteins), except for the anchovy *Engraulis encrasicolus*, with two proteins corresponding to different lineages or cryptic species [23]. The number of DNA sequences (haplotypes) per protein variant, as an indicator of the degree of subjacent genetic diversity, ranged from a value of 2 for the Amazonian sample to a value of 4.25 haplotypes per polypeptide for the Cantabric catch (Table 3), further highlighting the low genetic diversity in the exploited Amazonian fish examined here.

Limited genetic diversity was not associated with low biodiversity of the analyzed Amazonian catch. Ecological, taxonomic and phylogenetic diversity indices, as well as haplotype network complexity, reflect the exploited biodiversity as the number of species caught combined with their evolutionary interrelations. Biodiversity indices (ecological, taxonomic and phylogenetic) of the Amazonian catch were the highest of the four case studies considered (Table 3). The Amazonian haplotype network was also the most complex (Fig. 1R) because it contained more species belonging to different families, with considerably high distances between sequences. This network contained more internal nodes (21, in red) separating haplotypes, while the shape of the Mediterranean and Cantabric marine networks exhibited only six and four internal nodes respectively, indicating that these haplotypes were connected by less mutational steps and were phylogenetically closer than Amazonian genetic variants. Low metagenomic diversity despite high biodiversity can be considered a strong signal of depleted genetic diversity at the community level, and emphasizes the urgency of revising management of Amazonian fisheries.

## Discussion

The examples presented in this study reveal the potential of Barcoding data for rapid evaluation of diversity and, in a larger scope, for comparative studies of genetic diversity in different ecological settings [17]. Although DNA barcoding may not be sufficient to rigorously address population-level questions [24], it may be an ideal tool for early detection of genetic depletion of exploited species.

Fisheries overexploitation could be a possible cause of apparently reduced genetic diversity in the Amazonian catch [13], although likely not the only one. The patterns of commercial landings suggest overexploitation of Amazonian fisheries because large high-valued species declined significantly and were replaced by smaller, short-lived and lower-valued species [20]. Lower trophic level of Amazonian catch could be interpreted as an evidence of ‘fishing down the food web’ [25] and thus further independent evidence that Amazonian fisheries are overexploited. However, fishing pressure could not be the only possible reason for biodiversity loss. It is a well known fact that marine ecosystems possess higher levels of diversity than freshwater [e.g. 26], [27]; therefore, the differences found between the Amazon and the marine fisheries here analyzed could be a natural consequence of such ecosystem differences. Finally, this is an exploratory study and, as such, a limited number of sequences have been analyzed from each fishery. The possibility of a sampling artefact can not be excluded for explaining relatively low intra-specific diversity in Amazon samples, as well as biases in ecological, taxonomic and phylogenetic indices.

Treating Barcoding data [10], [11] like Eukaryote metagenomes will lead to a better understanding of the biodiversity exploited by humans. The importance of this type of metagenome-like approach becomes greater as long as the two levels of diversity are closely interrelated [4]. It could be applied not only to fishery science but also to a vast variety of ecological studies. We understand that further statistical and theoretical developments will be needed, and that the case studies depicted here are a simple example of the potential of this novel idea. Considering other genes of different degrees of conservation, large SNP datasets, and even whole genomes will surely be the next steps for analysing and understanding eukaryote metagenomes.

## Materials and Methods

The number of species inventoried from each ecosystem in our study was taken from FishBase (www.fishbase.org). The same database was consulted to obtain the trophic level of the species considered.

For each region, a total of 40 COI sequences were obtained from random samples of the species that represent 65% of the catch in each region, as estimated from the official regional catch statistics that can be found at http://www.ibama.gov.br/recursos-pesqueiros, http://www.fao.org, http://tematico.asturias.es/dgpesca/din/estalonj.php and http://tematico.asturias.es/mediambi/siapa/index.php for Amazonian (Manaus, Brazil), Mediterranean (Perpignan (Gulf of Lyon), Roussillon, France), Cantabric and north Spanish rivers (Narcea, Sella and Cares rivers; and different fish markets of Asturian region) respectively. Samples were obtained directly from local markets (or from fishermen in the case of north Spanish rivers, where sport catches are destined for personal consumption), sampled at random during at least five different weeks in 2010. In the case of Spanish rivers, Atlantic salmon catches are registered but the data for brown trout catch are based on surveys to anglers, being less reliable; by this reason we have expanded the sampling to >90% catch and included Atlantic salmon. The number of samples of each species was proportional to the relative weight of those species in the catch. The Amazonian sequences were obtained in the context of the Barcoding project [19].

Ecological (Shannon H), taxonomic (TTD) and phylogenetic (s*Φ* ^+^) indices of each regional catch (always the main species corresponding to 65% of the total catch in tons) were calculated using PRIMER 6 (Software package from Plymouth Marine Laboratory, UK). The total taxonomic distinctness TTD was applied because these communities are spatially independent and may vary in their phylogenetic composition [28]. s*Φ* ^+^ is the total variance of pairwise path lengths and can be interpreted as an index of the complexity of the hierarchical tree.

For obtaining the sequences we have employed the primers and methodology described by Ward et al [29]. Sequencing was performed with the DNA sequencing service GATC Biotech. Sequences were visualized and edited employing the BioEdit Sequence Alignment Editor software [30]. Sequences were aligned with the ClustalW application [31] included in BioEdit.

Conventional measures such as haplotype diversity have been employed because they potentially provide useful information in regard of the study of genetic diversity of species [12]. Sequence (nucleotide) diversity for each regional catch was estimated employing the DnaSP software [32], considering all sequences together without separating species.

The phylogenetic analysis was performed with the software MEGA 4.0 [33]. This software was also used to infer the putative protein (amino acid sequence) obtained from each COI sequence, and to construct the phylogenetic trees based on those amino acid sequences. The neighbor-joining (NJ) methodology was applied for phylogenetic inference, as is common in DNA barcoding studies [10]. The best suited model of protein sequence evolution and accompanying evolutionary parameter values for the data were determined using the PROTTEST [34], [35]. The best-fit evolutionary model of the amino acid sequences analyzed was JTT Matrix (Jones-Taylor-Thornton) [36], [37], with a gamma shape value of 4.59 for Amazonian, 4.60 for Cantabric Sea and 0.27 for Mediterranean Sea samples. Robustness of the NJ topology was assessed using 2,000 bootstrap replicates. Haplotypes networks were constructed with the program Network 4.5.1.6 (http://www.fluxus-engineering.com), with default settings.
